# A General Approach for Estimating Projective IRT Models

**DOI:** 10.1177/01466216261474955

**Published:** 2026-08-13

**Authors:** R. Philip Chalmers, Carl F. Falk, Steve P. Reise

**Affiliations:** 1 Department of Psychology, York University, Toronto, ON, Canada; 2 5620 Department of Psychology, McGill University, Montreal, QC, canada; 3 Department of Psychology, 158084 University of California Los Angeles, Los Angeles, CA, USA

**Keywords:** projective IRT, MIRT, bifactor models, MML, EM algorithm

## Abstract

Projective Item Response Theory (PIRT) modeling is a measurement framework in which multidimensional item response models are expressed in terms of lower-dimensional (e.g., unidimensional) proxy IRT models. Existing PIRT methods and their associated estimators currently rely on logistic function approximations that are limited to a narrow class of ordered, monotonic response functions. Though unexplored to date, these methods also require computationally intensive procedures to obtain sampling variability estimates for their resulting PIRT estimates. These and other limitations restrict the practical utility and broad application of PIRT models, particularly in empirical settings where only moderate sample sizes are available. To address these challenges, we introduce a general maximum marginal likelihood PIRT (MML-PIRT) approach that leverages components of the expectation–maximization (EM) algorithm commonly used in MML estimation. The proposed method uses expected count information generated during the EM-MML to fit proxy response functions for any focal trait of interest, and for a much broader class of multidimensional IRT models. In addition, MML-PIRT provides accurate and efficient large-sample variability estimates of the resulting PIRT model, thereby enhancing both the flexibility and statistical efficacy of PIRT model applications.

Item response theory (IRT) has become a predominant statistical framework for modeling response data commonly found in psychological measurement instruments, offering sophisticated tools for scale construction, evaluation, maintenance, and scoring. Central to the application of most operational IRT models, at least historically, is the assumption of unidimensionality (Meijer & Tendeiro, 2018; Reckase, 2009). This assumption posits that a single latent trait (*θ*) sufficiently explains the systematic co-variation among responses to the test items. Once the primary trait is controlled for, responses to items are assumed to be statistically independent, a condition known as local or conditional independence (Lord & Novick, 1968).

Although attractive in terms of mathematical, computational, and conceptual convenience, psychological data are rarely ever unidimensional. Educational and psychological tests often capture multiple latent variables, where systematic variation related to so-called “method” effects are common, constructs based on various “nuisance” test-taking strategies are present, interactions between multiple constructs exist, and so on. Hence, despite the attractiveness of forcing a unidimensional model onto data that is inherently multidimensional, doing so will negatively affect the model-data fit due to the inherent misspecification, leading to violations of local independence, biased parameter estimates, and, most importantly, can (sometimes severely) misrepresent the individuals being measured (Ackerman, 1989; Reckase et al., 1988; Reise et al., 2024). Recent work by Reise et al. (2024) further highlights the nature of this bias, demonstrating that while point estimates of the latent trait values can be robust to violations of unidimensionality, the misspecified factor loadings and resulting standard errors of those estimates are often unreasonably compromised.

Addressing known or suspected multidimensionality can often be addressed by fitting suitable multidimensional IRT (MIRT) models instead of the more optimistic unidimensional IRT (UIRT) models (Bock & Gibbons, 2021; Reckase, 2009). MIRT models come in various configurations for modeling between- and within-item factor loading patterns, and with various functional forms that connect the latent traits of interest to the observed response data. One model in particular that has seen resurgence in popularity, particularly because of its complementary relationship to unidimensional response models, is the bifactor model (Gibbons et al., 2007; Reise, 2012; Schmid & Leiman, 1957). The bifactor model’s factor structure partitions the covariance among items into a *general factor*, typically representing the construct of interest, and believed to be the dominant trait in the test, and one or more orthogonal *specific factors* representing nuisance dimensionality or sub-domains that are often of secondary importance.

Unfortunately, while MIRT models such as the bifactor model provide superior model-data fit to complex response data—sometimes to a fault (see Bonifay et al., 2025; Reise et al., 2016)—they often present substantive, interpretational, and application-based challenges (Li & Savalei, 2025). In part, these issues arise because of the nature of the specific factors modeled, which are often less conceptually and statistically reliable to interpret, and whose presence creates challenges when the primary goal of the test is to capture variation in the dominant general factor. Consequently, researchers are often left with a MIRT model that fits the response data well, but whose parameters hold less conceptual utility.

## Projective Item Response (PIRT) Models

As a potential solution to relying directly on MIRT models in applications, Projective Item Response Theory (PIRT) models have appeared as a framework that bridges the gap between the reality of multidimensional data and the interpretability of unidimensional models. Rather than assuming the response data are unidimensional at the outset, a PIRT model is obtained after first fitting an appropriate MIRT model to the respective items and subsequently projecting the higher-dimensional structure onto one or more lower dimensions (Ip, 2010a; Stucky et al., 2012). The PIRT model itself reflects a proxy model representation of the projected function, and therefore provides a convenient quantification of the single dimension of interest by first conditioning on the specific factors (i.e., nuisance effects) and subsequently marginalizing over the known contribution of these factors. Hence, the resulting PIRT model can be understood as the approximate (but accurate) response behavior caused by the dominant factor(s) averaged over the response behavior caused by the specific factors.^1^

Reise et al. (2024) highlight several attractive features of PIRT models, not just in terms of their conceptual clarity or practical solution to known conceptual complications (e.g., managing testlet effects; Wainer et al., 2007), but in terms of how these models can be used for population comparison purposes. In particular, the authors highlight how PIRT models can potentially be used for evaluating measurement invariance, test scoring, and how they can be used as a suitable diagnostic method for practitioners wishing to score a multidimensional assessment tool as though it were unidimensional (Strachan et al., 2020). Additionally, by comparing PIRT models to a strictly UIRT model, test analysts can empirically evaluate the practical consequences of ignoring known multidimensionality.

### Limitations

Despite the attractiveness of PIRT models, their widespread application has partially been hampered by their inflexibility and lack of efficient means to obtain sampling variability estimates of the resulting PIRT parameters. Specifically, PIRT implementations currently rely on a small set of response models belonging to the Multidimensional Two/Three-Parameter Logistic (M2PL/M3PL) and Multidimensional Graded Response Model (MGRM) families only (Doebler & Doebler, 2022; Ip, 2010a; Stucky et al., 2012), despite the fact that several MIRT models are in widespread use. As discussed further in the next section, the proposed logistic approximation approaches also require additional computational resources to approximate the sampling variability of the respective PIRT estimates—an area that has yet to be explicitly explored for PIRT models—and must apply a second strong assumption about the distribution of the latent traits and their associated scaling for the purpose of marginalization. Taken together, these limitations restrict researchers who are interested in utilizing PIRT models with other MIRT model families, more complex data structures, models with a large number of latent traits, MIRT models with scales that are not in a standardized metric, and when PIRT models are estimated from datasets with small to moderate sample sizes.

The purpose of the present work is to therefore address these and other limitations by introducing a novel PIRT estimation approach for any MIRT model that can be estimated using the Expectation–Maximization (EM) algorithm with the Maximum Marginal Likelihood (MML; Bock & Aitkin, 1981) criterion, with or without integration reduction techniques (Cai, 2010; Gibbons et al., 2007; Gibbons & Hedeker, 1992). This method, termed the MML-PIRT method, aims to overcome the limitations of current estimators and their associated proxy model representations, broadening the applicability of PIRT to a wider array of psychometric scenarios, and avoids the demanding computational steps required to obtain suitable standard errors of the resulting PIRT estimates.

## Obtaining PIRT Estimates Through Logistic Approximations

Beginning with MIRT models suitable for dichotomous response data, the multidimensional two-parameter logistic model (M2PL; Reckase, 1997) was initially investigated for PIRT modeling by Ip (2010a). The M2PL model with *D* + 1 traits is expressed in equation (1), though due to the PIRT context is reorganized such that a single latent trait (*θ*) is factored out of the complete set of latent traits being modeled, and the remaining *D* latent traits (Θ) are treated as nuisance or specific factors to be later marginalized (cf. Gibbons & Hedeker, 1992; Holzinger & Swineford, 1937; Horn, 1963). The probability of a positive endorsement (*y* = 1; where *y* ∈ {0, 1}) for the M2PL is

(1) $$P\left(y=1|\theta ,\Theta \right)=\frac{\exp\left(a\theta +a^{\prime }\Theta +d\right)}{1+\exp\left(a\theta +a^{\prime }\Theta +d\right)}$$

where *a* is the slope associated with the trait of interest, **a**′ is the vector of slopes for the *D* nuisance factors, and *d* is the item intercept.

For the resulting PIRT model the general response behavior caused by *θ* is of primary interest. To isolate the *θ*, the MIRT model is integrated over the *D* nuisance dimensions given an assumed density function, *g*(Θ) (typically, a multivariate normal distribution), resulting in the target function

(2) $$\bar{P}\left(y=1|\theta \right)=\int_{\Theta }P\left(y=1|\theta ,\Theta \right)g\left(\Theta \right)d\Theta .$$

Provided that the dimensionality of Θ is not too high, equation (2) can be obtained using precise numerical integration methods. However, it is also convenient to express the approximate behavior of this marginalized function using proxy PIRT models, in which case the logistic family can provide reasonably accurate parametric reflections of the function’s kernel (Ip, 2010a; Johnson & Kotz, 1970).

Focusing now on the logistic kernel approximation (LKA) approach explored by Ip (2010a, 2010b), which was constructed with the purpose of obtaining an empirically indistinguishable PIRT model containing the local dependencies implied by the MIRT model, the parametric approximation of equation (2) utilizes a logistic kernel that resembles a nested unidimensional version of the M2PL,

(3) $$\bar{P}\left(y=1|\theta \right)\approx \frac{\exp\left(\alpha^{*}\left(\theta -\beta^{*}\right)\right)}{1+\exp\left(\alpha^{*}\left(\theta -\beta^{*}\right)\right)}=\frac{\exp\left(a^{*}\theta +d^{*}\right)}{1+\exp\left(a^{*}\theta +d^{*}\right)},$$

where the slope-intercept parameterization is obtained from the discrimination-difficulty parameters via d* = −α*β* and a* = α*. In the case where there is only one nuisance dimension per item to marginalize, which appears in bifactor models (Gibbons & Hedeker, 1992), **a**′ in the M2PL reduces to the scalar *a*_*s*_, and the projected parameters are obtained using

$$\alpha^{*}=\lambda_{\mathrm{logit}}\left(a+\frac{a_{s}\rho \sigma_{s}}{\sigma }\right),\;\beta^{*}=-\frac{\lambda_{\mathrm{logit}}d}{\alpha^{*}}$$

where *ρ* is the population correlation between the latent traits (*ρ* = 0 for bifactor models), *σ*_*n*_ and *σ* are the standard deviation of the nuisance and primary trait of interest, respectively, $\lambda_{\mathrm{logit}}={\left[k^{2}a_{n}^{2}\left(1-\rho^{2}\right)\sigma_{n}^{2}+1\right]}^{-1/2}$ , and $k=\frac{16\sqrt{3}}{15\pi }$ . See Section 5.2 in Ip (2010a) for more general formula when marginalizing over multiple (potentially correlated) nuisance dimensions.

### PIRT for Multidimensional Graded Response Models

While the LKA approach proposed by Ip (2010a) was initially limited to M2PL (and M3PL) models this has not halted progress in obtaining other PIRT model variants, at least for ordered polytomous response data. In a similar spirit, Stucky et al. (2012) were the first to propose an alternative, factor analytic logistic model approximation relevant for ordered response data with the multidimensional graded response model (MGRM; Muraki & Carlson, 1995; Samejima, 1969). See also the later generalization of Ip’s LKA approach by Doebler and Doebler (2022) for MGRMs, which results in practically identical PIRT parameters as the following factor analytic method.

The MGRM is obtained through successive differences between ordered M2PL functions,

(4) P(y=t|θ,Θ)=P(y≥t−1|θ,Θ)−P(y≥t|θ,Θ),

where each slope coefficient associated with their respective latent trait is constrained to be equal across the *t* = 1, 2, …, *T* categories. Stucky et al.’s (2012) factor analytic approximation method, which for convenience we refer to as FA-PIRT, obtains the projected unidimensional GRM kernel approximation for this MGRM by manipulating the standardized loadings implied by the MIRT model (Bock et al., 1988). The method begins by converting the slope associated with the *θ* of interest (*a*) into a standardized factor loading (Bock & Aitkin, 1981; Bock et al., 1988). Next, this standardized loading is re-normalized in the factor loading space after removing the loadings associated with the nuisance factors, and finally back-transformed into the (now projected) proxy unidimensional GRM parameters.

Using the notation presented thus far, FA-PIRT therefore requires

$$\lambda_{a}=\frac{a/C}{\sqrt{1+{\left(a/C\right)}^{2}+\left(a^{\prime }a\right)/C^{2}}},\;\sigma_{a}=\sqrt{1-\lambda_{a}},$$

where *C* = 1.702. The *λ*_*a*_ and *σ*_*a*_ terms are then used to convert back to the logistic metric to approximate the kernel of (2) using

$$\alpha^{*}=\frac{\lambda_{a}}{\sigma_{a}}C,\;\beta_{t}^{*}=-\frac{d_{t}}{a}.$$

Again, a* = α* and $d_{t}^{*}=-\beta_{t}^{*}\alpha^{*}$ expresses the relationship between the canonical GRM parameterization and the slope-intercept version. See Reise et al. (2024) for worked demonstrations of these computations specifically in the context of bifactor models.

### Sample-Based Estimates of PIRT Parameters

One initially attractive feature of Stucky et al. (2012), as well as Ip’s (2010a) approach to the M2PL/M3PL and MGRM, is the availability of easy to use plug-in formula to obtain the logistic approximation parameters a* and $d_{t}^{*}$ (or equivalently, α* and $\beta_{t}^{*}$ ) given only the parameters in the M2PL/M3PL or MGRM themselves. Ip (2010a) demonstrated that these easy to use formulae provide optimal estimates of the logistic kernel functions that are often very close approximations to the true $\bar{P}\left(y=1|\theta \right)$ when evaluated with high-precision numerical integration methods. Similar precision can also be demonstrated for FA-PIRT and Doebler and Doebler’s (2022) LKA extension as well, resulting in logistic approximations that are, for all intents and purposes, interchangeable (see the simulation section below for direct comparison).

**Figure 1.**
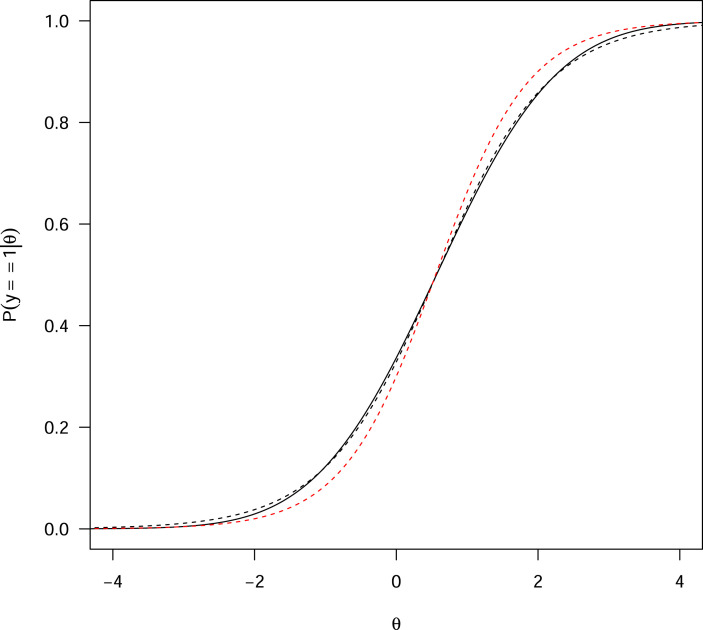
Comparison of marginal trace-lines for M2PL model. Solid black line reflects the true marginal population function obtained using numerical integration, the dashed black line the best fitting 2PL approximation using a least-squares criteria, and the dashed red line corresponds to a single sample estimate

Unfortunately, and as the following example highlights, the plug-in methods do not provide a natural means of obtaining sampling variability estimates of the resulting PIRT models, and do not provide a means to evaluate whether the LKA parameters for the associated proxy model can be improved upon in a given sample. Suppose that the parameters associated with a two-factor M2PL model were $a^{\prime }=\left[\begin{array}{cc} a_{1} & a_{2} \end{array}\right]$ , with *a*_1_ = 2.63, *a*_2_ = 1.63, and *d* = −1.49. In this case, Ip’s (2010a) LKA projection of the PIRT parameters results in the parameter vector ***ψ*** * = [a* d*] where a* = 1.274 and d* = −0.721. As initially observed in Ip (2010a), this approximation is indeed close to the true $\bar{P}\left(y=1|\theta \right)$ obtained through numerical integration (see Figure 1), deviating at most by Δ*P* ≈ 0.011, which occurs around *θ* ≈ − 0.24.

Had this marginal response function been available directly, however, then according to a least-squares definition of “best-fitting,” where

(5) $${\arg\;\min}_{\psi^{*}}\int_{\theta }{\left(\bar{P}\left(y=1|\theta \right)-P\left(y=1|\theta ,\psi^{*}\right)\right)}^{2}d\theta ,$$

a more optimal set of parameters could have been obtained; specifically the values a* = 1.260 and d* = −0.713. Using these least-squares PIRT parameters instead, the maximum absolute deviation becomes Δ*P* ≈ 0.008 across the range of *θ*. Evidently, while the logistic approximation to the marginal response function is indeed effective^2^, and of course remains useful in practice, this specific approximation may not reflect the most optimal approximation for the select proxy model when more direct information pertaining to the shape of $\bar{P}\left(y=1|\theta \right)$ is available.

Relatedly, the previous LKA presentations focused exclusively on the PIRT parameter transformations in terms of population-level behavior rather than sample-based behavior. In practice, only estimates of the parent model parameters are available, where the parent model is commonly estimated using (dimension reduced) maximum marginal likelihood (MML) or Bayesian estimation methods (Bock & Aitkin, 1981; Cai, 2010; Gibbons et al., 2007). As such, any deviations in the approximations from the true marginal PIRT function in the population may be further exacerbated due to random sampling, where less informative sample sizes ought to be accompanied by SE estimates to quantify this associated uncertainty.

Continuing with the above example, suppose the MML estimates of the M2PL model parameters were estimated to be ${\hat{a}}_{1}=3.064$ , ${\hat{a}}_{2}=2.964$ , $\hat{d}=-1.701$ for a dataset with *N* = 500 observations. Using the LKA plug-in formulas, the resulting PIRT estimates now become ${\hat{a}}^{*}=1.525$ and ${\hat{d}}^{*}=-0.846$ . This sample-based marginal function estimate is plotted as a dashed red line in Figure 1, where the largest deviation in this particular sample from the true function now appears around *θ* ≈ 1.536 with a maximum difference of approximately $\Delta \hat{P}=0.052$ . This generally highlights the importance of considering sampling variability when obtaining PIRT estimates as uncertainty in the PIRT estimates will lead to less optimal $\bar{P}\left(y=1|\theta \right)$ representations. To our knowledge, there has been no PIRT presentation that has addressed the sampling variability of the resulting PIRT estimates, despite the clear importance of doing so in practice.

## PIRT Estimates Using Components From MML Estimation

As it stands, no general-purpose PIRT modeling method has appeared that is agnostic to the type of MIRT model under investigation, nor have there been attempts at quantifying parametric functional forms other than logistic proxy model approximations of the projected response function. Moreover, to our knowledge there have been no publications that directly involve quantifying the sampling uncertainty of the marginal response function estimates, which is particularly problematic as the resulting estimates may be highly variable when applied to finite sample data.

To address these and other practical limitations, this section presents an approach that utilizes consequential information from the expectation–maximization (EM) algorithm for estimating MIRT model parameters (Bock & Aitkin, 1981; Dempster et al., 1977) for any combination of dichotomous or polytomous response data. As will be shown, this approach is particularly useful when applied to dimension reduction methods involving bifactor (Gibbons et al., 2007; Gibbons & Hedeker, 1992) and two-tier structures (Cai, 2010) as the computational constraints are limited only to the parent model, and can even avoid the issue of quantifying the sampling variability of the parent model itself. Note that because PIRT models are desirable for bifactor-type structures (Ip & Chen, 2012; Reise et al., 2024; Stucky et al., 2012) this presentation limits the focus to bifactor models with M2PL and MGRM response models. However, this is not a limitation of the proposed method, where in fact any proxy IRT functional form or parent model that be expressed within the EM-MML framework could be substituted without loss of generality.

### Bifactor Models With the EM-MML

Drawing from Gibbons et al. (2007), who presented a dimension-reduced MML estimation method with an EM algorithm suitable for bifactor models with polytomous items, we begin by defining the marginal response probability for response vector **y**_*i*_ for a test containing *J* items and *D* + 1 latent traits; one general dimension (*θ*) and *D* independent specific/nuisance dimensions (**Θ**). The bifactor model implies that the marginal probability for the response vector **y**_*i*_ is

(6) $$P\left(y=y_{i}|\Psi \right)=\int_{\theta }\left\{\overset{D}{\underset{s=1}{\prod }}\int_{\Theta_{s}}L_{is}\left(\theta ,\Theta_{s},\Psi \right)g\left(\Theta_{s}\right)d\Theta_{s}\right\}g\left(\theta \right)d\theta$$

where

$$L_{is}\left(\theta ,\Theta ,\Psi \right)=\overset{J}{\underset{j=1}{\prod }}\overset{T_{j}}{\underset{t=1}{\prod }}\Phi_{jt}{\left(\theta ,\Theta ,\Psi \right)}^{\mu_{js}x_{\mathrm{ijt}}}$$

reflects the associated likelihood function given the category item response function, Φ_*jt*_(⋅). In the above equations, *x*_*ijt*_ is 1 when item *j* within response pattern *i* for category *t* is observed (and 0 otherwise), *T*_*j*_ is the number of categories for the *j*th item, Ψ is a *p*-dimensional vector of parameters to be estimated, and *μ*_*js*_ is 1 when there is a non-zero loading of item *j* on dimension *s* and 0 otherwise.

#### Expectation (E-Step)

With the dimension reduction approach there are at most two integrals to evaluate in any bifactor model specification, which simplifies the subsequent numerical integration demand when the marginal likelihood is required (cf. Bock & Aitkin, 1981). When forming the table of expected counts suitable for the E-step of the EM algorithm, which is required with or without the above dimension reduction approach, the weight function

(7) $$E_{is}\left(\theta ,\Psi \right)=\frac{\left[\prod_{h=1}^{D}\int_{\Theta_{h}}L_{ih}\left(\theta ,\Theta_{h},\Psi \right)g\left(\Theta_{h}\right)d\Theta_{h}\right]}{\int_{\Theta_{s}}L_{is}\left(\theta ,\Theta_{s},\Psi \right)g\left(\Theta_{s}\right)d\Theta_{s}}$$

is also needed. When applied to sample data the above equations are evaluated using discrete quadrature, where all *g*(⋅) probability density functions are typically assumed to be from the Gaussian family.

In the context of the EM algorithm, the computational benefit of using discrete quadrature is that it allows a table of expected counts to be populated whenever the observed-data log-likelihood in equation (6) is evaluated. Specifically, this table of expected counts is obtained by

(8) $${\bar{r}}_{\mathrm{jts}}\left(X_{q},X_{q_{s}},\Psi \right)=\frac{\sum_{i=1}^{N}x_{\mathrm{ijt}}\left[E_{is}\left(X_{q},\Psi \right)\right]L_{is}\left(X_{q},X_{q_{s}},\Psi \right)}{P\left(y=y_{i}|\Psi \right)},$$

where ${\bar{r}}_{\mathrm{jts}}$ represents the expected number of responses for category *t* in item *j*, summed over all *N* response patterns, and *X*_*q*_ and $X_{q_{s}}$ are the discrete quadrature associated with the primary and nuisance dimensions, respectively. Note that analogous presentations are available when using the traditional EM approach described in Bock and Aitkin (1981), as well as in Cai’s (2010) two-tier modeling approach, depending on how the table of expected counts is formed.

#### Maximization (M-Step)

Given the expected table of counts in (8), the M-step is then performed by treating this information as provisionally fixed. The requisite M-step complete-data log-likelihood for item *j* is

(9) $$L_{j}\left(\Psi \right)=\overset{Q}{\underset{q=1}{\sum }}\overset{Q}{\underset{q_{s}=1}{\sum }}\overset{T_{j}}{\underset{t=1}{\sum }}{\bar{r}}_{\mathrm{jts}}\left(X_{q},X_{q_{s}},\tilde{\Psi }\right)\log\Phi_{jt}\left(X_{q},X_{q_{s}},\Psi \right)$$

where *q* = 1, *…*, *Q* and *q*_*s*_ = 1, *…*, *Q* index quadrature nodes for the general and relevant specific dimension, respectively, and ${\bar{r}}_{\mathrm{jts}}\left(X_{q},X_{q_{s}},\tilde{\Psi }\right)$ are expected counts at some interim value of the model parameters, $\tilde{\Psi }$ , such as from a previous EM iteration.

#### EM-MML Algorithm

Finally, the EM algorithm is executed by starting with some provisional $\Psi =\hat{\Psi }$ , computing the table expected counts from (8), and subsequently updating $\hat{\Psi }$ in the M-step by simultaneously maximizing (9) for all *J* items. This latter step often uses probit or logit regression solutions for the response functions under investigation (Bock & Aitkin, 1981). The algorithm then repeats the two-step sequence until the updated $\hat{\Psi }$ converges to the MML solution, $\hat{\Psi }={\hat{\Psi }}^{\left(MLE\right)}$ , where the algorithm is terminated early if the size of the successive $\hat{\Psi }$ updates is sufficiently small, changes between the observed log-likelihood are minimal, or if a maximum number of iterations is reached.

### The MML-PIRT Approach

Once the final estimate of $\hat{\Psi }$ has been obtained, and capitalizing on the fact that the E-table already contains the numerically integrated expected count information, subsequent conditional expectation count information suitable for PIRT models can naturally be obtained for any dimension of interest by simply summing over the nuisance $X_{q_{s}}$ terms. Manipulation of the E-table for modeling purposes is a common strategy in the IRT literature; for instance, this topic appears in contexts that involve estimating the shape or moments of the underlying *θ* distribution (Bock & Aitkin, 1981; Bock & Zimowski, 1997; Mislevy, 1984; Woods, 2011), when investing differential item response functioning in multiple-group contexts (Thissen et al., 1988), when computing effect sizes for differential test functioning (Chalmers, 2018a, 2022), and so on. As such, it should come as no surprise that summation operations involving the E-table are relevant in the context of PIRT models as well.

Following standard probability laws for marginalization, the unidimensional PIRT for any bifactor model requires summing across the E-table to marginalize the effects of the specific latent traits, resulting in *marginal* expected count tables (ME-tables),

(10) $${\bar{r}}_{jt}\left(X_{q},\hat{\Psi }\right)=\overset{Q}{\underset{q_{s}=1}{\sum }}{\bar{r}}_{\mathrm{jts}}\left(X_{q},X_{q_{s}},\hat{\Psi }\right).$$

Applying the summation in (10) will obtain the expected counts for each of the *X*_*q*_ quadrature on the first dimension only as this composite directly marginalizes the contribution of the nuisance dimensions. This logic can be further generalized to the multi-factor modeling case, whereby the marginalization is performed over all *D* dimensions that are not of interest

(11) $${\bar{r}}_{jt}\left(X_{q},\hat{\Psi }\right)=\overset{Q}{\underset{q_{1}=1}{\sum }}\overset{Q}{\underset{q_{2}=1}{\sum }}\cdots \overset{Q}{\underset{q_{D}=1}{\sum }}{\bar{r}}_{\mathrm{jts}}\left(X_{q},X_{q_{1}},X_{q_{2}},\ldots ,X_{q_{D}},\hat{\Psi }\right).$$

This summation logic may be applied to portions of the E-table when multiple traits are of interest, creating potential for multidimensional PIRT models, or further marginalized to obtain estimates of the projected *g*(*θ*) functions; however, these topics are not explored in the current presentation. Finally, the marginalized table of counts in equation (10) or (11) can be used to write the projective M-step log-likelihood for item *j*,

(12) $$L_{j}\left(\Psi^{*}\right)=\overset{Q}{\underset{q=1}{\sum }}\overset{T_{j}}{\underset{t=1}{\sum }}{\bar{r}}_{jt}\left(X_{q},\hat{\Psi }\right)\log\Phi_{jt}\left(X_{q},\Psi^{*}\right)$$

which, in contrast to equation (9), only requires summation over quadrature nodes for the general dimension as the relevant specific dimensions have already been marginalized. Maximizing equation (12) completes the MML-PIRT implementation, resulting in the estimated ${\hat{\Psi }}^{*}$ vector.

Conceptually, the M-step portion of the MML-PIRT method treats the ME-table as though it were the canonical E-table with the EM algorithm in the MML application. However, unlike the canonical E-table, the ME-table should *not* be updated after obtaining the estimates of the resulting PIRT model as this would remove the likelihood constraints implied by the parent MIRT model. That is, ${\bar{r}}_{jt}\left(X_{q},\hat{\Psi }\right)$ is computed only once based on estimates from the parent model, and optimization of equation (12) is required only once for all items.

One initial benefit of the MML-PIRT implementation is that any proxy response function capable of adequately representing the behavior depicted in the marginal response table may now be fitted to obtain estimates of the PIRT parameters. With respect to which functional form to use on the ME-table, the most natural candidates are still those that match the kernel of the projection function (e.g., the 2PL for the M2PL with a unidimensional projection, the GRM for the MGRM, etc.), however these need not be the only functions to select. For complex MIRT model projections, for example, it may be advantageous to select more flexible response models, such as monotonic spline (Ramsay & Winsberg, 1991) or monotone polynomial models (Falk & Cai, 2016), while projections into more than one dimension may require fitting similar MIRT models (e.g., fitting the M2PL with only two dimensions when MML-PIRT marginalizes all but two dominant factors). Another advantage is that direct modeling of non-normal distributions has direct and natural consequences on the resulting PIRT estimates; however, unlike the LKA and FA methods, the MML-PIRT approach does not need to re-introduce the latent variable distribution information at a later stage.

Taken together, and regardless of whether the original E-table was obtained using dimension reduction, the subsequent ME-table provides a direct projection of the expected response behavior implied by the fitted MIRT model, behaving in the same manner as an E-table obtained from a constrained lower-dimensional IRT model. Here, the constrained ME-table is based on the (typically MLE) parameter estimates and the observed sample information for the fitted MIRT model, thereby providing suitable model-based information for estimating the shape and associated sampling uncertainty of the proxy response function—the latter of which is described in the next section.

### Sampling Variability for PIRT Estimates

After locating a suitable $\hat{\Psi }={\hat{\Psi }}^{\left(MLE\right)}$ vector in the parent MIRT model, and subsequently obtaining the projective estimates ${\hat{\Psi }}^{*}$ , the resulting PIRT estimates can be understood as belonging to a location on the resulting lower-dimensional likelihood surface that is somewhere (potentially far) from the highest mode of the projected likelihood function^3^. Given this information, it remains possible—and indeed, desirable—to compute the (expected) second-order Taylor series expansion around the constrained ${\hat{\Psi }}^{*}$ to obtain the observed or expected information matrix, $I\left({\hat{\Psi }}^{*}\right)$ , which is akin to locating the sampling variability components found in the likelihood Score test (Pawitan, 2001).

When this resulting matrix is inverted this provides the matrix $\Sigma_{{\hat{\Psi }}^{*}}=I^{-1}\left({\hat{\Psi }}^{*}\right)$ , which can be used in subsequent inferential operations, including the computation of SEs for the PIRT parameter estimates (Pawitan, 2001). Note that although the PIRT vector is clearly not at the mode of the lower-dimensional likelihood function, which SEs are typically derived from, $\Sigma_{{\hat{\Psi }}^{*}}$ is asymptotically equivalent to the inverted information matrix evaluated at the mode of this likelihood function and can therefore be used as a suitable stand-in (Van Der Vaart, 1998). Importantly, with this approach estimating the asymptotic covariance matrix for the parent model, $\Sigma_{\hat{\Psi }}$ , is not a prerequisite. This is fortunate as $\Sigma_{\hat{\Psi }}$ is often significantly more prohibitive to obtain due to the higher dimensionality of the parent model (Cai et al., 2011; Chalmers, 2018b; Liu & Chalmers, 2021).

In contrast to the direct estimation of $\Sigma_{{\hat{\Psi }}^{*}}$ with MML-PIRT, the related logistic approximation methods derived by Ip (2010a), Doebler and Doebler (2022), and Stucky et al. (2012)—although not evaluated by the original authors—require estimates of the full sampling variability of the parent model, $\Sigma_{\hat{\Psi }}$ , in order to obtain relevant sampling variability implied by $\Sigma_{{\hat{\Psi }}^{*}}$ . This parent-model sampling variability can be estimated using a range of approaches, each of which involves distinct trade-offs in precision and computational burden. For example, the most straightforward likelihood-based approximation is to apply the delta method to $\Sigma_{\hat{\Psi }}$ , combined with an appropriate Jacobian of the transformation, to derive the associated large-sample $\mathrm{SE}\left({\hat{\Psi }}^{*}\right)$ estimates. Alternatively, albeit with substantially greater computational cost, one may re-estimate the parent model using Markov chain Monte Carlo (MCMC) algorithms, or use parametric and non-parametric bootstrapping (Efron & Tibshirani, 1998) to approximate the complete posterior distribution.

Regardless of the select approach suitable for the logistic approximation approaches, methods that require the sampling variability of the parent model will naturally be more computationally intensive than MML-PIRT, particularly because the latter does not require estimation of the parent model’s sampling variability. Nevertheless, these more involved methods can be advantageous in small-sample settings, where $\Sigma_{{\hat{\Psi }}^{*}}$ derived under large-sample likelihood theory may provide overly optimistic characterizations of the sampling variability.

## Comparing PIRT Estimation Methods

This section provides a brief comparison of the competing PIRT estimators with two simulated data examples. The first example utilizes information found in Reise et al. (2024) when fitting bifactor models to dichotomous response data with the M2PL, while the second example explores a similar bifactor application with the MGRM presented by Gibbons et al. (2007) for polytomous response data.

### M2PL Example

Beginning with a M2PL application, Reise et al. (2024) presented several types of potential bifactor structures to demonstrate the properties and associated computations of the PIRT logistic kernel approximations discussed earlier. To demonstrate how MML-PIRT compares, the information presented in their Table 3 (p. 351) was adopted and augmented to obtain suitable sample data. See Table 1 for the population parameters for the primary dimension (*a*), the nuisance factor (*a*_*Grp*_), and intercepts (*d*), as well as the associated parameter estimates obtained after generating sample data with *N* = 10,000 response vectors from a set of latent trait values drawn from independent standard normal distributions.^4^

**Table 1.** Parameters adopted from Table 3 in Reise et al. (2024), augmented to include intercept parameters and their resulting estimates on a sample generated with *N* = 10, 000 observations. Large-sample standard errors for the parameter estimates are included in parentheses

	Population generating parameters					Estimated parameters				
	*A*	*a*_*Grp*1_	*a*_*Grp*2_	*a*_*Grp*3_	*d*	$\hat{a}$	${\hat{a}}_{\mathrm{Grp1}}$	${\hat{a}}_{\mathrm{Grp2}}$	${\hat{a}}_{\mathrm{Grp3}}$	$\hat{d}$
1	2.63	3.07			−1.49	2.73 (.10)	2.85 (.11)			−1.57 (.07)
2	2.63	3.07			1.08	2.71 (.10)	2.99 (.11)			0.93 (.06)
3	2.63	3.07			−0.93	2.54 (.09)	2.92 (.11)			−0.95 (.06)
4	2.63	3.07			3.04	2.57 (.11)	3.07 (.13)			3.01 (.11)
5	2.63	3.07			1.60	2.58 (.09)	2.90 (.10)			1.48 (.06)
6	1.63		1.36		1.48	1.65 (.06)		1.47 (.07)		1.60 (.05)
7	1.63		1.36		0.04	1.64 (.05)		1.52 (.07)		0.08 (.03)
8	1.63		1.36		1.01	1.58 (.05)		1.42 (.06)		0.97 (.04)
9	1.63		1.36		0.86	1.67 (.05)		1.41 (.06)		0.89 (.04)
10	1.63		1.36		1.63	1.59 (.05)		1.33 (.06)		1.37 (.04)
11	1.38			0.69	−2.32	1.36 (.05)			0.74 (.10)	−2.35 (.06)
12	1.38			0.69	1.53	1.36 (.05)			0.71 (.09)	1.50 (.04)
13	1.38			0.69	0.23	1.37 (.04)			0.70 (.08)	0.21 (.03)
14	1.38			0.69	−0.99	1.36 (.04)			0.65 (.08)	−1.03 (.03)
15	1.38			0.69	−1.49	1.40 (.05)			0.79 (.09)	−1.45 (.04)

For the PIRT parameters, the population a* and d* terms were obtained by fitting a 2PL function directly to the integrated marginal function using the least-squares criteria in (5). These resulting parameters therefore slightly differ from what is obtained in Reise et al. (2024), as these parameters correspond to the least-square variants of the 2PL model fitted to the marginal probability functions as opposed to the logistic kernel approximation. The MML-PIRT with a fitted 2PL model, and the two analytic logistic approximation methods, were used to obtain the PIRT estimates ${\hat{a}}^{*}$ and ${\hat{d}}^{*}$ presented in Table 2, along with their associated large-sample SEs from the estimated $\Sigma_{\hat{\Psi }}$ (via the delta method) and $\Sigma_{{\hat{\Psi }}^{*}}$ (for MML-PIRT). However, because Ip's (2010a) and Stucky et al.’s (2012) FA-IRT results were identical up to the third decimal place only Ip’s method is presented.

**Table 2.** PIRT estimates and the population a* and d* parameters based on fitting a least-square 2PL model to the numerically integrated marginal probability function. Standard error are presented in parentheses

	a*	${\hat{a}}_{\text{LKA}}^{*}$	${\hat{a}}_{\text{MML}-\text{PIRT}}^{*}$	d*	${\hat{d}}_{\text{LKA}}^{*}$	${\hat{d}}_{\text{MML}-\text{PIRT}}^{*}$
1	1.26	1.40 (0.05)	1.37 (0.04)	−0.71	−0.80 (0.03)	−0.79 (0.03)
2	1.26	1.34 (0.04)	1.31 (0.04)	0.51	0.46 (0.03)	0.45 (0.03)
3	1.26	1.28 (0.04)	1.24 (0.04)	−0.44	−0.48 (0.03)	−0.46 (0.03)
4	1.26	1.25 (0.05)	1.25 (0.04)	1.45	1.46 (0.03)	1.45 (0.03)
5	1.26	1.31 (0.04)	1.28 (0.04)	0.76	0.75 (0.03)	0.73 (0.03)
6	1.25	1.25 (0.05)	1.24 (0.04)	1.14	1.21 (0.03)	1.19 (0.03)
7	1.25	1.22 (0.04)	1.18 (0.04)	0.03	0.06 (0.03)	0.06 (0.03)
8	1.25	1.21 (0.04)	1.19 (0.04)	0.77	0.75 (0.03)	0.73 (0.03)
9	1.25	1.28 (0.05)	1.26 (0.04)	0.66	0.68 (0.03)	0.67 (0.03)
10	1.25	1.26 (0.05)	1.24 (0.04)	1.04	1.08 (0.03)	1.06 (0.03)
11	1.26	1.25 (0.06)	1.26 (0.05)	−2.13	−2.16 (0.05)	−2.16 (0.04)
12	1.26	1.25 (0.05)	1.25 (0.04)	1.40	1.39 (0.03)	1.37 (0.03)
13	1.26	1.27 (0.05)	1.25 (0.04)	0.21	0.20 (0.03)	0.19 (0.03)
14	1.26	1.28 (0.05)	1.27 (0.04)	−0.91	−0.96 (0.03)	−0.96 (0.03)
15	1.26	1.27 (0.05)	1.26 (0.04)	−1.37	−1.32 (0.03)	−1.31 (0.03)

**Figure 2.**
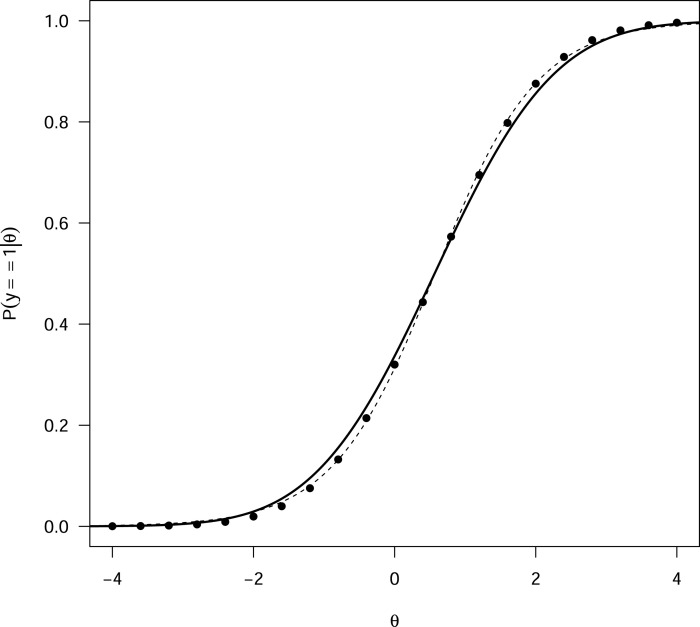
Comparison of the marginal trace-line obtained via numerical integration (solid line) and the estimates obtained using the implied marginal E-table proportions (points) and the associated 2PL function estimate (dashed line)

To highlight the logic of the MML-PIRT approach, Figure 2 depicts the population PIRT function for Item 1, as estimated using the least-squares criterion, against the resulting MML-PIRT and discrete empirical proportions from the ME-table, computed at each quadrature. As is clear from inspection, the empirical proportions, and subsequently fitted MML-PIRT model, indeed reflect the properties of the population PIRT model, deviating only slightly due to sampling variability.

With respect to the recovery of the population a* and d* terms, all PIRT estimation methods appeared to recover the respective parameters reasonably well; however, the MML-PIRT approach on average appeared to be slightly closer to the population parameters according to the estimated bias and root mean-square deviation (RMSD) behavior. With respect to a* and d*, using either Ip's (2010a) or the FA-PIRT approach resulted in RMSD estimates of .047 and .042, respectively, while for the MML-PIRT these estimates were .042 and .040. To determine whether this trend was consistent across other samples, and whether this trend is present across different sample sizes, a later section evaluates this described example using Monte Carlo experimentation.

### MGRM Example

As a second working example, the MML-PIRT and FA-IRT method were applied to a set of ordered polytomous response data that were generated according to the reported MGRM parameters in Gibbons et al. (2007), Table 1 (p. 11), where *a*_*Grp*_ indicates each unique specific factor’s slope. Due to the larger size of this model the online supplement contains the associated MGRM population generating parameters, the population values of the least-squares solutions for the resulting PIRT model, and all associated parameter estimates. Note that in this example the related LKA method described in Doebler and Doebler (2022) was omitted as the results were redundant with the results from FA-PIRT.

Sample data were drawn to have *N* = 578 to match the associated article, where again the latent traits were assumed to be from independent standard normal distributions. However, note that the “Global” item was structured to have no specific factor slope parameter as in the original article this term reflected only a singlet item, loading on only one dimension. Hence, for this example *a*_*Grp*_ = 0 for the “Global” item, and was not freely estimated in the parent model. As such, this reflects a situation where the resulting PIRT model, when fitted with the same functional form as the parent model, will have identical parameters and sample estimates.

Beginning with the parent bifactor model, Table S2 in the online supplement contains the parameter estimates and associated SEs returned from the *mirt* package (Chalmers, 2012), generally reflecting accurate recovery of the population generating model. However, given the smaller sample size compared to the previous M2PL example, and the larger number of parameters required, wider SEs and larger deviations from the population model were observed. Evidently, given the higher degree of parameter uncertainty it is important to include SE estimates of the resulting PIRT model as the sampling uncertainty in the parent model ultimately leads to uncertainty in the resulting PIRT model.

The PIRT estimates and SEs from the FA-PIRT and MML-PIRT methods were indeed quite similar, as depicted in online supplement tables, generally highlighting the strong agreement of the PIRT estimators. However, for these sample data, the MML-PIRT was generally superior to the FA-PIRT approach in terms of parameter recovery. For the a* terms, FA-IRT resulted in an RMSD of 0.098, while for MML-PIRT the RMSD was 0.086, while all SE(a*) estimates were lower for the MML-PIRT, also reflecting lower sampling variability. A similar trend appeared for the respective d*_k_ terms, where for FA-PIRT the RMSD for d*_1_ through d*_6_ were [0.083, 0.083, 0.086, 0.084, 0.099, 0.142], while for MML-PIRT only half of the RMSD value were lower, [0.076, 0.079, 0.086, 0.082, 0.089, 0.135], and approximately 87% of the $\mathrm{SE}\left(d_{k}^{*}\right)$ were lower with the MML-PIRT method than the FA-IRT. Evidently, the PIRT estimators capitalize on different characteristics of the sample data, where in this case MML-PIRT appeared slightly superior to the FA-IRT approach (and related LKA approach by Doebler & Doebler, 2022) in terms of parameter recovery and estimated sampling precision.

After the bifactor and subsequent PIRT model are constructed they may be used to predict the individual general factor locations by utilizing either the complete vector of responses or, more conveniently (though less precisely), using suitable sum-score conversion look-up tables (Thissen et al., 1995; Thissen & Wainer, 2001). For bifactor models where sum-scores are deemed practically useful, the modified Lord-Wingersky algorithm described by Cai (2015) can be used to marginalize the specific factors prior to obtaining the general factor predictions associated with each sum-score. This process is intimately related to how the PIRT model itself is conceptualized, though applied to test scoring instead, and is generally preferable to using the PIRT model directly for scoring as PIRT models will result in more liberal SE(*θ*) behavior. That said, scoring individuals using either the updated Lord-Wingersky algorithm applied to the bifactor directly, or using the original Lord-Wingersky algorithm with the PIRT model, will be better than using the misspecified UIRT model due to the ignored local dependencies (Reise et al., 2024).

As an alternative to scoring the complete item set directly, a suitable short-form PIRT model capable of capturing variation in the general trait could be constructed by including exactly one item from each specific factor. The reason for selecting only one item per specific factor is because the product of marginal likelihood functions—which are required when computing *θ*_*i*_ predictions—are generally not equivalent to the product of conditional likelihood functions implied by bifactor model (Stucky et al., 2012). Selecting only one item from each factor to build a short-form test avoids this local dependence issue, and can provide an unbiased indication of what the general-factor prediction would have been had the full test been administered.

One potentially optimal item subset suitable for building a short-form PIRT model can be obtained by selecting items based on which specific factor item demonstrates the largest projected $\left|{\hat{a}}^{*}\right|$ value (in this case, Global, Family 4, Finance 3, …, Social 2). This eight-item short-form would contain maximal marginal discrimination properties for the general factor, thereby providing a reasonable amount of precision for the general factor of interest^5^. Interested readers can refer to Table S5 in the online supplement, which depicts the measurement properties of this particular short-form for the MGRM example above by way of the Expected a Posteriori (EAP) sum-score predictions (Thissen et al., 1995) for the 49 possible sum-scores, resulting in a marginal reliability estimate for the primary dimension of .756.

## Simulation Experiments

Given the competitive nature of the PIRT estimators, a small set of Monte Carlo simulations were explored using the population generating models presented in the previous examples. Specifically, this section utilizes the same parameters and analysis methods as presented earlier, however evaluates these examples across four sample sizes, *N* = [500, 1000, 5000, 10000]. Each simulation condition was repeated across *R* = 1000 independent replicates and subsequently summarized in terms of their bias and RMSD behavior relative to the more computationally intensive population least-squares slope and intercept terms, as well as their general ability to recover the shape of the true marginal response function obtained through numerical integration (equation (2)).

With respect to the MML-PIRT approach, UIRT proxy models were fitted to the ME-tables to serve as a direct comparison to the competing logistic approximation methods. As well, the discrete proportion components of the ME-table (i.e., the discrete point estimates in Figure 2) were studied to estimate the general convergence of the non-parametric estimate of the standardized table of expected counts, where recovery of the slope and intercept parameters is inapplicable in this context.

For all methods studied, the comparison between the integrated marginal response function and the estimated proxy versions of this function were obtained using

$$RMSD-Q=\sqrt{\frac{\sum_{q=1}^{Q}{\left(\bar{P}\left(X_{q}\right)-\hat{P}\left(X_{q}\right)\right)}^{2}}{Q}}$$

where *Q* is the number of evenly spaced discrete quadrature (*X*_*q*_) within the interval [−6, 6], $\bar{P}\left(X_{q}\right)$ is the *j*th item marginal probability function obtained by numerical integration given the population parameters, evaluated at *X*_*q*_, and $\hat{P}\left(X_{q}\right)$ the functional estimate from the discrete or logistic estimated response function evaluated at *X*_*q*_. Note that *Q* was fixed to 31 as by default this is the total number of quadrature per dimension used for the bifactor model estimation in *mirt* (Chalmers, 2012). Complete simulation results and code organized with the *SimDesign* package (Chalmers & Adkins, 2020) are available upon request.

### Simulation for M2PL

Simulation results pertaining to the parameter recovery of the least-squares a* and d* terms are found in Table 3. In general, and again as expected, the LKA and FA-PIRT approaches were nearly identical, demonstrating slightly higher bias in smaller sample sizes for the a* parameter and similar RMSD behavior across all sample sizes. In contrast, MML-PIRT demonstrated lower bias for the a* terms, and in general demonstrated lower bias overall, however this estimator provided marginally less efficient parameter recovery of a* in the *N* = 500 and *N* = 1000 conditions. Nevertheless, the differences were very minor, suggesting the general equivalence of these estimators for this particular M2PL data generation and proxy model.

**Table 3.** Marginal recovery behavior of the slopes and intercepts for the three logistic approximation approaches

*N*	Parameter	Bias			RMSD		
		FA-PIRT	LKA	MML-PIRT	FA-PIRT	LKA	MML-PIRT
500	a*	0.034	0.034	0.018	0.228	0.228	0.229
1000		0.023	0.022	0.007	0.157	0.157	0.158
5000		0.017	0.017	0.001	0.070	0.070	0.070
10000		0.016	0.015	−0.001	0.050	0.050	0.050
500	d*	0.006	0.006	0.002	0.147	0.147	0.147
1000		0.008	0.007	0.002	0.102	0.102	0.102
5000		0.005	0.005	−0.001	0.046	0.046	0.045
10000		0.004	0.004	−0.002	0.033	0.033	0.032

With respect to the general recovery of the marginal response functions obtained via integration as seen in Table 4, the recovery at the select *Q* quadrature locations reflected that the marginal function was recovered with greater precision as the sample sizes increase. As well, the three estimators that fitted the logistic proxy model were nearly identical at recovering the true marginalized function across the sample sizes, highlighting that while the parameter estimates may slightly differ in the individual samples the general recovery behavior of the marginal function is effectively equivalent. The exception to this trend was the MML-PIRT with empirical proportions, which demonstrated slightly less optimal recovery in the smaller sample sizes but greater precision as sample size increased.

**Table 4.** RMSD-Q behavior compared to the function $\bar{P}\left(\theta \right)$

*N*	FA-PIRT	LKA	MML-PIRT	MML-PIRT (empirical)
500	0.028	0.028	0.028	0.030
1000	0.019	0.019	0.019	0.021
5000	0.009	0.009	0.009	0.009
10000	0.007	0.007	0.007	0.006

Taken together, the estimators explored for the M2PL were generally interchangeable in terms of their parameter recovery, where none of the estimators appeared to be a globally superior choice, and all of which performed well in terms of recovering the shape of the true $\bar{P}\left(\theta \right)$ . In the context of this presentation, this simulation provides beneficial support with respect to adopting the MML-PIRT estimator as a general purpose PIRT estimation method.

### Simulation for MGRM

Continuing with the MGRM example, the following simulation experiment followed the same data generation and analyses process as the M2PL simulation above. To save space, however, the PIRT intercept parameter recovery information was marginalized to provide a less cluttered presentation of the recovery behavior.

Beginning with the recovery of the projected GRM parameters, as seen in Table 5 the plug-in methods (FA-PIRT, and Doebler and Doebler’s LKA generalization) again demonstrated slight bias in the parameter recovery across the sample sizes studied, where a* was recovered with an estimated bias around .02, while MML-PIRT generally did not demonstrate any notable bias effects. Similarly, the plug-in methods showed slightly negative bias in the estimation of the intercept parameters, where again MML-PIRT did not. Additionally, although all estimators improved as sample size increased, MML-PIRT demonstrated equivalent or, in some cases, improved parameter recovery efficacy compared to the plug-in methods, particularly for the a* parameters.

**Table 5.** Marginal recovery behavior of the slopes and (average) intercepts for the logistic approximation approaches

*N*	Parameter	Bias			RMSD		
		FA-PIRT	LKA	MML-PIRT	FA-PIRT	LKA	MML-PIRT
500	a*	0.022	0.021	0.003	0.114	0.114	0.112
1000		0.021	0.020	0.002	0.083	0.083	0.080
5000		0.018	0.018	0.000	0.041	0.041	0.036
10000		0.018	0.017	−0.001	0.032	0.032	0.026
500	${\bar{d}}_{k}^{*}$	−0.003	−0.003	0.003	0.126	0.125	0.124
1000		−0.009	−0.008	−0.003	0.093	0.093	0.091
5000		−0.006	−0.006	0.000	0.052	0.051	0.049
10000		−0.006	−0.006	0.000	0.043	0.043	0.041

With respect to recovering the seven category trace-lines in the projected GRM, though averaging over these category trace-lines for presentation purposes, Table 6 demonstrates the general exchangeability in the plug-in methods and the MML-PIRT estimator. In this case, however, the empirical proportion method was notably less optimal than either proxy modeling method, primarily because the expected counts across the seven response categories were small relative to the total *N*, leading to sparseness issues in the resulting proportions. Hence, for polytomous response data it will often be more beneficial to focus on the parametrically smoothed versions of the PIRT functions instead of the empirical proportions when focusing directly on the category response functions—as well as any resulting item score function (Embretson & Reise, 2000)—unless the sample sizes are extremely large.

**Table 6.** Average RMSD-Q behavior compared to the $\bar{P}\left(\theta \right)$ functions for the unidimensional GRM

*N*	FA-PIRT	LKA	MML-PIRT	MML-PIRT (empirical)
500	0.017	0.017	0.017	0.111
1000	0.012	0.012	0.012	0.090
5000	0.006	0.006	0.005	0.054
10000	0.004	0.004	0.004	0.042

## Discussion

The present article introduced a general Maximum Marginal Likelihood-Projected IRT (MML-PIRT) approach suitable for obtaining PIRT parameter estimates for any parent multidimensional IRT model that can be accommodated by standard MML routines with the EM algorithm, and with efficient and accurate likelihood-based sampling variability estimates. Unlike existing approaches that rely on logistic approximations for limited classes of multidimensional IRT models, the proposed method directly leverages the expected-table of counts generated at the final iteration of the EM algorithm to estimate the parameters for any PIRT function of interest, and can naturally quantify the degree of sampling variability in these associated parameter estimates.

As was discussed throughout, MML-PIRT can be applied to any MIRT model that can be expressed within the EM-MML framework, with or without dimension reduction techniques for higher-dimensional models, and for any type of proxy model or projected dimension of interest. As such, although bifactor models projected onto unidimensional spaces pertaining to the general dimension were presented, this need not be the only application as MML-PIRT could be used in other projective IRT contexts, such as in two-tier IRT models (Cai, 2010) with more than one general factor to create a marginalized version of a “simple structure” PIRT model (Thurstone, 1954), or when projecting to the specific factors instead to create PIRT models suitable for sub-score investigations (Haberman et al., 2024). Finally, and unique to the MML-PIRT method, obtaining sampling variability estimates of the parent MIRT model is not required for estimating the resulting sampling variability of the resulting PIRT model parameters, and other modeling utilities such as including parameter equality and inequality constraints are naturally supported, both of which offer a substantial advantage over previous logistic approximation methods involving plug-in formulae.

The simplicity and computationally efficiency of the present method is also beneficial as MML-PIRT is straightforward to implement in existing IRT software. Specifically, any software capable of estimating MIRT models using the EM-algorithm can adopt the MML-PIRT method as all of the necessary computational components are already available—including, for instance, fitting nested lower dimensional UIRT or MIRT models to the projected ME-table. As a demonstration of this, the function *pirt*() has been added directly to the *mirt* package (Chalmers, 2012) to automate such analyses. Using this function, users can pass their fitted MIRT models to perform the projective IRT methods described in this article, along with the described SE estimation methods. Overall, the ease of integration to existing software is attractive in that auxiliary functions for plotting, person scoring, diagnostic methods, and more may be explored whenever PIRT models are deemed useful.

### Limitations and Future Directions

Notwithstanding the strengths of the MML-PIRT approach, several limitations and opportunities for extension warrant discussion. First, although the method makes use of expected-count tables that arise naturally during MML estimation with the EM algorithm, the computational burden will often increase with the dimensionality of the parent MIRT model and the granularity of the integration grid—particularly for MIRT models that are not amenable to dimension reduction techniques. Second, although the simulation results generally indicate that the MML-PIRT is competitive with (and in many cases, superior to) prior PIRT logistic approximation methods, the simulation results are nowhere near conclusive. Indeed, due to the nature of the ME-table it would not be surprising to observe alternative inferential behavior whenever the expected counts are sparce, and when more flexible IRT models are fitted to the projected ME-table. Fortunately, Bayesian priors can be used in the proxy models as well, which would be particularly useful for more flexible IRT models (e.g., Ramsay & Winsberg, 1991), though more work is required in this area to completely understand these intricacies.

Third, the evaluation of projected item response functions invites new diagnostic tools. Because the projected response surfaces provide a complete representation of the relationship between the focal trait and expected item responses, analysts may consider using these surfaces to: construct localized misfit indices (e.g., Stone, 2000); compare projected and empirical trace lines to detect marginal assumption violations, similar to how Q–Q plots are used for residuals in regression modeling; gauge the robustness of the resulting PIRT model (Strachan et al., 2020); and so on. In this sense, the projective parameter estimates serve not only as a bridge between multidimensional and constrained unidimensional modeling, but also as a potential diagnostic instrument that may enhance substantive interpretation. Future work should explore the MML-PIRT for models not presented in this article as well, such as the multidimensional nominal response model (Thissen et al., 2010) and related divide-by-total models, multidimensional ideal point models (Maydeu-Olivares et al., 2006), exploratory factor analysis models (Bock et al., 1988), Thurstone IRT models (Brown & Maydeu-Olivares, 2013), partially and fully non-compensatory response models (Chalmers, 2020), among others.

Lastly, though not exhaustively, the MML-PIRT framework can naturally be extended to more complex modeling contexts, including multidimensional models with covariates, testlet structures, differential item functioning investigations, longitudinal and multilevel modeling designs, and more. Extensions to Bayesian estimation are also be feasible by substituting posterior-mean expected counts from stochastic samplers to replace the deterministic MML E-tables required in the EM algorithm (Diebolt & Ip, 1996), which may be a particularly attractive area when quadrature-based integration methods become too restrictive. Such extensions would further broaden the utility of the PIRT framework for applied psychological measurement, particularly for educational and psychological assessment, clinical outcomes measurement, and large-scale survey research.

## Supplemental Material

Supplemental Material - A General Approach for Estimating Projective IRT Models Supplemental Material for A General Approach for Estimating Projective IRT Models by R. Philip Chalmers, Carl F. Falk, Steve P. Reise in Applied Psychological Measurement.

## Notes

1. Similar applications are possible in linear factor analysis models as well, however the projection operations will not require numerical integration as the projections are exact (e.g., via a Schur-complement; see also the Frisch-Waugh-Lovell theorem). While linear approximations could be useful in the IRT context, perhaps a means to obtain starting values for iterative methods, we restrict our focus to projections involving non-linear functions.

2. The least-squares and LKA methods overlapped so much in fact that they could not be diserned in the presented graphic, and therefore only the least-squares version was plotted.

3. Had the mode of this projection function been the target of the optimization this would result in fitting a standard (but misspecified) IRT model to the original data to obtain the MLEs, such as when fitting the 2PL model to data reflecting a M2PL structure. This property implies that a likelihood ratio test could be evaluated between the constrained PIRT parameter set and the unconstrained MLE parameter set, though this is unlikely to be directly useful as the PIRT model will necessarily fit worse than the MLE in the projected likelihood space.

4. Sample data were generated and fitted using the *mirt* package (Chalmers, 2012), where $\Sigma_{\hat{\Psi }}$ and $\Sigma_{{\hat{\Psi }}^{*}}$ were estimated using the Oakes identity method (Chalmers, 2018b).

5. More generally, one would want to inspect the Fisher information functions for *θ* implied by the PIRT model for selecting an optimal short-form to ensure the measurement information is sufficiently spread (or concentrated) at particular *θ* locations as measurement information is also a function of the projected intercepts.
