# Supplementary material for: A General Approach for Estimating Projective IRT Models
Source: Appl Psychol Meas. 2026 Aug 13:01466216261474955. Online ahead of print. doi: 10.1177/01466216261474955 (PMC13473346; doi:10.1177/01466216261474955)
Supplement: Supplementary file 1 [file sj-pdf-1-apm-10.1177_01466216261474955.pdf]

Online Supplement

On-line supplement for the article "A General Approach for Estimating Projective IRT Models" containing the parameters and parameter estimates for the MGRM and associated PIRT models.

Table 1: Parameters adopted from Table 1 in Gibbons et al. (2007), with the associated PIRT parameters from the least-squares criterion.

	Population IRT Model								Projected Parameters						
	a	a_{Grp}	d_1	d_2	d_3	d_4	d_5	d_6	a^*	d_1^*	d_2^*	d_3^*	d_4^*	d_5^*	d_6^*
Global	1.64	—	1.00	0.46	0.05	-0.45	-1.27	-2.19	1.64	1.00	0.46	0.05	-0.44	-1.27	-2.19
Family 1	1.29	1.47	0.63	0.09	-0.32	-0.81	-1.63	-2.56	0.95	0.52	0.10	-0.22	-0.60	-1.21	-1.89
Family 2	1.26	1.05	1.05	0.51	0.10	-0.39	-1.21	-2.14	1.06	0.87	0.43	0.10	-0.32	-1.01	-1.80
Family 3	1.42	1.34	1.68	1.14	0.73	0.24	-0.58	-1.51	1.11	1.30	0.90	0.58	0.19	-0.46	-1.19
Family 4	1.60	1.32	1.04	0.51	0.10	-0.39	-1.22	-2.14	1.24	0.82	0.40	0.07	-0.32	-0.95	-1.66
Finance 1	1.06	1.38	1.60	1.07	0.66	0.16	-0.66	-1.58	0.80	1.22	0.81	0.50	0.13	-0.50	-1.20
Finance 2	0.85	1.03	1.26	0.72	0.31	-0.18	-1.00	-1.93	0.70	1.06	0.61	0.27	-0.15	-0.83	-1.61
Finance 3	1.30	1.46	1.71	1.18	0.77	0.28	-0.55	-1.47	0.97	1.29	0.89	0.58	0.21	-0.40	-1.09
Finance 4	1.27	1.46	1.64	1.10	0.69	0.20	-0.62	-1.55	0.93	1.18	0.77	0.45	0.09	-0.49	-1.17
Health 1	0.92	0.54	0.91	0.38	-0.03	-0.53	-1.35	-2.28	0.85	1.01	0.50	0.11	-0.38	-1.18	-2.07
Health 2	1.04	0.92	0.69	0.16	-0.25	-0.74	-1.57	-2.49	0.90	0.61	0.14	-0.21	-0.64	-1.35	-2.15
Health 3	0.93	0.84	0.95	0.42	0.01	-0.48	-1.31	-2.23	0.82	0.88	0.40	0.04	-0.40	-1.13	-1.96
Health 4	1.03	0.81	0.77	0.24	-0.17	-0.66	-1.49	-2.41	0.91	0.66	0.18	-0.18	-0.61	-1.33	-2.14
Health 5	1.21	0.65	0.81	0.28	-0.13	-0.63	-1.45	-2.38	1.11	0.75	0.26	-0.10	-0.55	-1.31	-2.18
Health 6	1.34	0.41	1.11	0.57	0.17	-0.33	-1.15	-2.08	1.30	1.08	0.56	0.16	-0.32	-1.11	-2.01
Leisure 1	1.39	0.60	1.26	0.72	0.31	-0.18	-1.00	-1.93	1.30	1.18	0.67	0.29	-0.17	-0.94	-1.80
Leisure 2	1.10	0.74	1.10	0.57	0.16	-0.34	-1.16	-2.08	0.99	1.00	0.51	0.14	-0.31	-1.05	-1.88
Leisure 3	1.37	0.91	0.84	0.31	-0.10	-0.60	-1.42	-2.34	1.20	0.71	0.22	-0.16	-0.60	-1.36	-2.11
Leisure 4	1.50	1.08	1.12	0.59	0.18	-0.31	-1.14	-2.06	1.24	0.94	0.50	0.16	-0.25	-0.93	-1.70
Leisure 5	1.18	0.89	1.09	0.55	0.14	-0.35	-1.17	-2.10	1.03	0.96	0.48	0.11	-0.31	-1.03	-1.84
Leisure 6	0.79	0.31	0.62	0.08	-0.33	-0.82	-1.64	-2.57	0.77	0.57	0.05	-0.35	-0.83	-1.64	-2.54
Living 1	1.17	1.17	0.96	0.42	0.01	-0.48	-1.30	-2.23	0.93	0.76	0.34	0.00	-0.38	-1.05	-1.78
Living 2	1.00	1.05	0.41	-0.12	-0.53	-1.03	-1.85	-2.78	0.83	0.35	-0.09	-0.45	-0.86	-1.55	-2.31
Living 3	1.29	1.64	0.69	0.15	-0.26	-0.75	-1.57	-2.50	0.90	0.48	0.10	-0.18	-0.53	-1.10	-1.75
Living 4	1.37	1.87	0.30	-0.23	-0.64	-1.13	-1.96	-2.88	0.94	0.03	-0.32	-0.58	-0.89	-1.41	-2.00
Living 5	1.29	1.73	1.29	0.76	0.35	-0.14	-0.97	-1.89	0.88	0.89	0.52	0.24	-0.10	-0.66	-1.29
Safety 1	1.18	1.03	1.10	0.56	0.15	-0.34	-1.16	-2.09	1.00	0.82	0.37	0.04	-0.35	-1.03	-1.79
Safety 2	1.26	0.97	0.73	0.19	-0.22	-0.71	-1.53	-2.46	1.07	0.67	0.20	-0.15	-0.58	-1.29	-2.09
Safety 3	1.09	0.96	1.33	0.80	0.39	-0.11	-0.93	-1.85	0.93	1.09	0.64	0.28	-0.13	-0.81	-1.59
Safety 4	1.22	1.10	1.18	0.64	0.24	-0.26	-1.08	-2.01	1.01	0.98	0.53	0.20	-0.21	-0.89	-1.66
Safety 5	1.16	0.71	0.86	0.32	-0.08	-0.58	-1.40	-2.33	1.05	0.79	0.30	-0.07	-0.52	-1.27	-2.12
Social 1	1.04	0.69	0.78	0.24	-0.16	-0.66	-1.48	-2.41	0.96	0.69	0.20	-0.15	-0.61	-1.37	-2.21
Social 2	1.08	0.54	0.98	0.45	0.04	-0.45	-1.28	-2.20	1.02	0.97	0.45	0.07	-0.41	-1.19	-2.08
Social 3	0.99	0.73	0.79	0.25	-0.15	-0.65	-1.47	-2.40	0.91	0.71	0.22	-0.15	-0.60	-1.36	-2.21
Social 4	0.73	0.42	0.56	0.02	-0.39	-0.88	-1.70	-2.63	0.70	0.54	0.04	-0.35	-0.82	-1.62	-2.51

Table 2: MGRM bifactor model parameter estimates and SEs (in parentheses) from MML estimation.

	$\hat{a}$	$\hat{a}_{Grp}$	$\hat{d}_1$	$\hat{d}_2$	$\hat{d}_3$	$\hat{d}_4$	$\hat{d}_5$	$\hat{d}_6$
Global	1.61 (0.13)	—	0.91 (0.12)	0.31 (0.12)	-0.16 (0.12)	-0.45 (0.12)	-1.30 (0.13)	-2.50 (0.17)
Family 1	1.50 (0.16)	1.47 (0.20)	0.66 (0.14)	0.17 (0.14)	-0.27 (0.14)	-0.82 (0.15)	-1.74 (0.18)	-2.75 (0.22)
Family 2	1.20 (0.12)	0.98 (0.14)	1.14 (0.13)	0.56 (0.12)	0.13 (0.12)	-0.39 (0.12)	-1.22 (0.13)	-1.95 (0.15)
Family 3	1.58 (0.15)	1.27 (0.17)	1.86 (0.17)	1.31 (0.15)	0.86 (0.14)	0.34 (0.14)	-0.60 (0.14)	-1.50 (0.16)
Family 4	1.80 (0.17)	1.35 (0.18)	1.00 (0.15)	0.44 (0.14)	0.02 (0.14)	-0.48 (0.15)	-1.50 (0.17)	-2.42 (0.20)
Finance 1	0.99 (0.11)	1.12 (0.13)	1.56 (0.14)	0.96 (0.12)	0.61 (0.12)	0.09 (0.11)	-0.76 (0.12)	-1.64 (0.14)
Finance 2	0.81 (0.11)	1.10 (0.13)	1.33 (0.13)	0.86 (0.12)	0.33 (0.11)	-0.14 (0.11)	-1.06 (0.12)	-2.08 (0.15)
Finance 3	1.30 (0.15)	1.57 (0.18)	1.71 (0.17)	1.17 (0.15)	0.79 (0.14)	0.42 (0.14)	-0.42 (0.14)	-1.40 (0.16)
Finance 4	1.56 (0.21)	2.30 (0.32)	2.35 (0.27)	1.58 (0.22)	0.93 (0.18)	0.38 (0.17)	-0.87 (0.18)	-2.00 (0.24)
Health 1	1.03 (0.10)	0.34 (0.12)	0.90 (0.11)	0.40 (0.10)	0.01 (0.10)	-0.53 (0.10)	-1.32 (0.12)	-2.38 (0.15)
Health 2	1.13 (0.12)	0.97 (0.17)	0.79 (0.12)	0.21 (0.12)	-0.22 (0.12)	-0.79 (0.12)	-1.68 (0.15)	-2.62 (0.19)
Health 3	0.89 (0.11)	0.92 (0.18)	1.11 (0.12)	0.56 (0.11)	0.14 (0.11)	-0.30 (0.11)	-1.26 (0.13)	-2.09 (0.16)
Health 4	1.07 (0.12)	1.08 (0.21)	0.99 (0.13)	0.43 (0.12)	-0.08 (0.12)	-0.65 (0.12)	-1.55 (0.15)	-2.37 (0.19)
Health 5	1.22 (0.11)	0.61 (0.14)	0.78 (0.12)	0.42 (0.11)	-0.02 (0.11)	-0.54 (0.11)	-1.31 (0.13)	-2.21 (0.15)
Health 6	1.43 (0.12)	0.37 (0.13)	1.14 (0.12)	0.58 (0.12)	0.18 (0.11)	-0.38 (0.11)	-1.23 (0.13)	-2.23 (0.15)
Leisure 1	1.57 (0.13)	0.75 (0.15)	1.42 (0.14)	0.80 (0.13)	0.29 (0.12)	-0.15 (0.12)	-1.02 (0.13)	-1.81 (0.15)
Leisure 2	1.07 (0.11)	0.71 (0.15)	1.03 (0.12)	0.44 (0.11)	0.04 (0.11)	-0.45 (0.11)	-1.29 (0.13)	-2.08 (0.15)
Leisure 3	1.61 (0.14)	0.91 (0.17)	0.78 (0.13)	0.23 (0.13)	-0.23 (0.13)	-0.79 (0.13)	-1.64 (0.15)	-2.62 (0.19)
Leisure 4	1.49 (0.14)	1.06 (0.19)	1.01 (0.14)	0.51 (0.13)	0.21 (0.13)	-0.26 (0.13)	-1.18 (0.14)	-2.07 (0.18)
Leisure 5	1.25 (0.12)	0.84 (0.16)	1.07 (0.13)	0.53 (0.12)	0.20 (0.12)	-0.36 (0.12)	-1.22 (0.13)	-2.16 (0.16)
Leisure 6	0.84 (0.10)	0.39 (0.12)	0.59 (0.10)	0.18 (0.10)	-0.24 (0.10)	-0.79 (0.10)	-1.52 (0.12)	-2.30 (0.15)
Living 1	1.22 (0.13)	1.23 (0.14)	1.06 (0.13)	0.57 (0.13)	0.25 (0.12)	-0.23 (0.12)	-1.18 (0.14)	-2.13 (0.16)
Living 2	1.18 (0.13)	1.24 (0.14)	0.57 (0.12)	0.13 (0.12)	-0.37 (0.12)	-1.01 (0.13)	-1.86 (0.15)	-2.78 (0.19)
Living 3	1.69 (0.18)	1.95 (0.21)	0.99 (0.17)	0.38 (0.16)	-0.17 (0.16)	-0.79 (0.16)	-1.73 (0.19)	-2.72 (0.23)
Living 4	1.49 (0.17)	1.85 (0.20)	0.41 (0.15)	-0.25 (0.15)	-0.62 (0.15)	-1.08 (0.16)	-1.95 (0.19)	-2.90 (0.23)
Living 5	1.13 (0.13)	1.30 (0.14)	1.14 (0.13)	0.65 (0.13)	0.41 (0.12)	-0.07 (0.12)	-0.81 (0.13)	-1.55 (0.14)
Safety 1	0.93 (0.11)	0.69 (0.14)	0.90 (0.11)	0.41 (0.10)	-0.02 (0.10)	-0.47 (0.11)	-1.06 (0.12)	-1.83 (0.14)
Safety 2	1.19 (0.12)	0.69 (0.14)	0.76 (0.12)	0.11 (0.11)	-0.24 (0.11)	-0.80 (0.12)	-1.56 (0.13)	-2.30 (0.16)
Safety 3	1.09 (0.12)	1.09 (0.19)	1.21 (0.14)	0.60 (0.12)	0.16 (0.12)	-0.33 (0.12)	-1.12 (0.14)	-2.04 (0.17)
Safety 4	1.27 (0.13)	0.96 (0.17)	1.13 (0.13)	0.65 (0.12)	0.34 (0.12)	-0.28 (0.12)	-1.20 (0.14)	-2.25 (0.17)
Safety 5	1.21 (0.12)	0.78 (0.15)	0.88 (0.12)	0.35 (0.11)	-0.03 (0.11)	-0.60 (0.12)	-1.44 (0.13)	-2.46 (0.17)
Social 1	1.01 (0.12)	0.88 (0.25)	0.75 (0.12)	0.29 (0.11)	-0.19 (0.11)	-0.63 (0.12)	-1.44 (0.15)	-2.38 (0.21)
Social 2	1.04 (0.11)	0.52 (0.17)	0.95 (0.11)	0.45 (0.11)	0.03 (0.10)	-0.51 (0.11)	-1.33 (0.12)	-2.20 (0.15)
Social 3	1.09 (0.12)	0.84 (0.24)	0.82 (0.12)	0.22 (0.11)	-0.11 (0.11)	-0.54 (0.12)	-1.65 (0.16)	-2.61 (0.21)
Social 4	0.73 (0.10)	0.52 (0.16)	0.47 (0.10)	-0.00 (0.10)	-0.43 (0.10)	-1.02 (0.11)	-1.71 (0.13)	-2.61 (0.17)

Table 3: Associated parameter estimates and delta method SEs (in parentheses) for the FA-IRT method.

	$\hat{a}^*$	$\hat{d}_1^*$	$\hat{d}_2^*$	$\hat{d}_3^*$	$\hat{d}_4^*$	$\hat{d}_5^*$	$\hat{d}_6^*$
Global	1.61 (0.13)	0.91 (0.12)	0.31 (0.12)	-0.16 (0.12)	-0.45 (0.12)	-1.30 (0.13)	-2.50 (0.17)
Family 1	1.13 (0.11)	0.50 (0.11)	0.13 (0.10)	-0.21 (0.10)	-0.62 (0.11)	-1.31 (0.12)	-2.08 (0.13)
Family 2	1.04 (0.10)	0.99 (0.11)	0.48 (0.10)	0.12 (0.10)	-0.34 (0.10)	-1.05 (0.11)	-1.69 (0.12)
Family 3	1.27 (0.11)	1.49 (0.12)	1.05 (0.12)	0.69 (0.11)	0.28 (0.11)	-0.48 (0.11)	-1.20 (0.12)
Family 4	1.41 (0.12)	0.78 (0.12)	0.34 (0.11)	0.02 (0.11)	-0.38 (0.11)	-1.17 (0.12)	-1.90 (0.14)
Finance 1	0.83 (0.09)	1.30 (0.11)	0.80 (0.10)	0.51 (0.10)	0.07 (0.10)	-0.64 (0.10)	-1.37 (0.11)
Finance 2	0.68 (0.09)	1.12 (0.10)	0.72 (0.10)	0.28 (0.09)	-0.12 (0.09)	-0.89 (0.10)	-1.75 (0.12)
Finance 3	0.96 (0.10)	1.25 (0.11)	0.86 (0.10)	0.58 (0.10)	0.31 (0.10)	-0.31 (0.10)	-1.03 (0.11)
Finance 4	0.93 (0.10)	1.40 (0.11)	0.94 (0.10)	0.55 (0.10)	0.22 (0.10)	-0.52 (0.10)	-1.19 (0.11)
Health 1	1.01 (0.10)	0.88 (0.11)	0.39 (0.10)	0.01 (0.10)	-0.52 (0.10)	-1.30 (0.11)	-2.33 (0.15)
Health 2	0.98 (0.10)	0.69 (0.10)	0.18 (0.10)	-0.19 (0.10)	-0.69 (0.10)	-1.46 (0.11)	-2.28 (0.14)
Health 3	0.79 (0.09)	0.98 (0.10)	0.49 (0.10)	0.12 (0.09)	-0.27 (0.10)	-1.11 (0.10)	-1.84 (0.12)
Health 4	0.90 (0.10)	0.84 (0.10)	0.36 (0.10)	-0.07 (0.10)	-0.55 (0.10)	-1.31 (0.11)	-2.00 (0.13)
Health 5	1.15 (0.11)	0.74 (0.11)	0.39 (0.10)	-0.01 (0.10)	-0.51 (0.11)	-1.23 (0.12)	-2.09 (0.14)
Health 6	1.40 (0.12)	1.12 (0.12)	0.57 (0.11)	0.17 (0.11)	-0.37 (0.11)	-1.20 (0.12)	-2.18 (0.15)
Leisure 1	1.44 (0.12)	1.30 (0.12)	0.73 (0.12)	0.27 (0.11)	-0.14 (0.11)	-0.93 (0.12)	-1.66 (0.13)
Leisure 2	0.99 (0.10)	0.95 (0.11)	0.41 (0.10)	0.04 (0.10)	-0.41 (0.10)	-1.20 (0.11)	-1.92 (0.13)
Leisure 3	1.42 (0.12)	0.68 (0.11)	0.20 (0.11)	-0.20 (0.11)	-0.70 (0.12)	-1.44 (0.13)	-2.31 (0.15)
Leisure 4	1.26 (0.11)	0.86 (0.11)	0.43 (0.11)	0.18 (0.11)	-0.22 (0.11)	-1.01 (0.11)	-1.76 (0.13)
Leisure 5	1.12 (0.11)	0.96 (0.11)	0.48 (0.10)	0.18 (0.10)	-0.32 (0.10)	-1.10 (0.11)	-1.93 (0.13)
Leisure 6	0.82 (0.10)	0.57 (0.10)	0.17 (0.09)	-0.23 (0.09)	-0.77 (0.10)	-1.48 (0.11)	-2.25 (0.14)
Living 1	0.99 (0.10)	0.86 (0.11)	0.46 (0.10)	0.20 (0.10)	-0.19 (0.10)	-0.96 (0.11)	-1.73 (0.12)
Living 2	0.96 (0.10)	0.46 (0.10)	0.11 (0.10)	-0.30 (0.10)	-0.82 (0.10)	-1.50 (0.11)	-2.25 (0.14)
Living 3	1.11 (0.11)	0.65 (0.11)	0.25 (0.10)	-0.11 (0.10)	-0.52 (0.10)	-1.14 (0.11)	-1.79 (0.12)
Living 4	1.00 (0.10)	0.28 (0.10)	-0.17 (0.10)	-0.42 (0.10)	-0.73 (0.10)	-1.32 (0.11)	-1.96 (0.13)
Living 5	0.90 (0.10)	0.91 (0.10)	0.51 (0.10)	0.33 (0.10)	-0.05 (0.10)	-0.64 (0.10)	-1.23 (0.11)
Safety 1	0.86 (0.10)	0.84 (0.10)	0.38 (0.10)	-0.02 (0.10)	-0.43 (0.10)	-0.98 (0.10)	-1.70 (0.12)
Safety 2	1.10 (0.11)	0.71 (0.11)	0.10 (0.10)	-0.22 (0.10)	-0.75 (0.11)	-1.44 (0.12)	-2.13 (0.14)
Safety 3	0.92 (0.10)	1.02 (0.11)	0.51 (0.10)	0.14 (0.10)	-0.27 (0.10)	-0.94 (0.10)	-1.72 (0.12)
Safety 4	1.10 (0.10)	0.99 (0.11)	0.57 (0.10)	0.30 (0.10)	-0.24 (0.10)	-1.05 (0.11)	-1.96 (0.13)
Safety 5	1.10 (0.11)	0.80 (0.11)	0.32 (0.10)	-0.02 (0.10)	-0.54 (0.10)	-1.31 (0.12)	-2.24 (0.14)
Social 1	0.90 (0.10)	0.67 (0.10)	0.26 (0.10)	-0.17 (0.10)	-0.56 (0.10)	-1.28 (0.11)	-2.11 (0.13)
Social 2	0.99 (0.10)	0.91 (0.11)	0.43 (0.10)	0.03 (0.10)	-0.49 (0.10)	-1.27 (0.11)	-2.10 (0.14)
Social 3	0.98 (0.10)	0.73 (0.10)	0.20 (0.10)	-0.10 (0.10)	-0.49 (0.10)	-1.48 (0.12)	-2.34 (0.14)
Social 4	0.70 (0.09)	0.45 (0.09)	-0.00 (0.09)	-0.41 (0.09)	-0.97 (0.10)	-1.64 (0.12)	-2.49 (0.15)

Table 4: Associated parameter estimates and SEs (in parentheses) for MML-PIRT.

	$\hat{a}^*$	$\hat{d}_1^*$	$\hat{d}_2^*$	$\hat{d}_3^*$	$\hat{d}_4^*$	$\hat{d}_5^*$	$\hat{d}_6^*$
Global	1.61 (0.13)	0.91 (0.12)	0.31 (0.12)	-0.16 (0.12)	-0.45 (0.12)	-1.30 (0.13)	-2.50 (0.17)
Family 1	1.11 (0.11)	0.49 (0.10)	0.14 (0.10)	-0.18 (0.10)	-0.57 (0.10)	-1.24 (0.11)	-2.02 (0.14)
Family 2	1.03 (0.10)	0.98 (0.11)	0.48 (0.10)	0.12 (0.10)	-0.33 (0.10)	-1.04 (0.11)	-1.67 (0.12)
Family 3	1.24 (0.11)	1.47 (0.12)	1.03 (0.11)	0.67 (0.11)	0.26 (0.11)	-0.48 (0.11)	-1.18 (0.12)
Family 4	1.37 (0.12)	0.76 (0.11)	0.33 (0.11)	0.01 (0.11)	-0.37 (0.11)	-1.14 (0.12)	-1.87 (0.14)
Finance 1	0.81 (0.09)	1.26 (0.11)	0.77 (0.10)	0.49 (0.10)	0.06 (0.09)	-0.63 (0.10)	-1.35 (0.11)
Finance 2	0.67 (0.09)	1.07 (0.10)	0.68 (0.09)	0.25 (0.09)	-0.11 (0.09)	-0.86 (0.10)	-1.71 (0.12)
Finance 3	0.93 (0.10)	1.22 (0.11)	0.83 (0.10)	0.57 (0.10)	0.31 (0.10)	-0.28 (0.10)	-0.99 (0.11)
Finance 4	0.91 (0.10)	1.34 (0.11)	0.90 (0.10)	0.53 (0.10)	0.22 (0.10)	-0.49 (0.10)	-1.15 (0.11)
Health 1	1.01 (0.10)	0.88 (0.10)	0.39 (0.10)	0.01 (0.10)	-0.52 (0.10)	-1.29 (0.11)	-2.33 (0.15)
Health 2	0.98 (0.10)	0.67 (0.10)	0.18 (0.10)	-0.19 (0.10)	-0.66 (0.10)	-1.42 (0.11)	-2.24 (0.14)
Health 3	0.77 (0.09)	0.95 (0.10)	0.47 (0.09)	0.11 (0.09)	-0.27 (0.09)	-1.09 (0.10)	-1.80 (0.12)
Health 4	0.89 (0.09)	0.81 (0.10)	0.34 (0.10)	-0.07 (0.10)	-0.53 (0.10)	-1.28 (0.11)	-1.97 (0.13)
Health 5	1.14 (0.11)	0.72 (0.11)	0.39 (0.10)	-0.01 (0.10)	-0.50 (0.10)	-1.20 (0.11)	-2.06 (0.14)
Health 6	1.39 (0.12)	1.12 (0.12)	0.58 (0.11)	0.18 (0.11)	-0.36 (0.11)	-1.19 (0.12)	-2.17 (0.15)
Leisure 1	1.43 (0.12)	1.28 (0.12)	0.72 (0.11)	0.26 (0.11)	-0.14 (0.11)	-0.92 (0.12)	-1.64 (0.13)
Leisure 2	0.99 (0.10)	0.95 (0.11)	0.41 (0.10)	0.05 (0.10)	-0.39 (0.10)	-1.17 (0.11)	-1.91 (0.13)
Leisure 3	1.40 (0.12)	0.67 (0.11)	0.18 (0.11)	-0.22 (0.11)	-0.70 (0.11)	-1.42 (0.13)	-2.27 (0.15)
Leisure 4	1.23 (0.11)	0.84 (0.11)	0.43 (0.11)	0.17 (0.11)	-0.22 (0.11)	-0.99 (0.11)	-1.73 (0.13)
Leisure 5	1.13 (0.10)	0.94 (0.11)	0.47 (0.10)	0.18 (0.10)	-0.32 (0.10)	-1.09 (0.11)	-1.92 (0.13)
Leisure 6	0.81 (0.09)	0.57 (0.10)	0.17 (0.09)	-0.23 (0.09)	-0.76 (0.10)	-1.46 (0.11)	-2.23 (0.14)
Living 1	0.95 (0.10)	0.84 (0.10)	0.45 (0.10)	0.21 (0.10)	-0.16 (0.10)	-0.92 (0.10)	-1.69 (0.12)
Living 2	0.92 (0.10)	0.46 (0.10)	0.12 (0.10)	-0.27 (0.10)	-0.78 (0.10)	-1.46 (0.12)	-2.22 (0.14)
Living 3	1.06 (0.10)	0.65 (0.10)	0.27 (0.10)	-0.08 (0.10)	-0.47 (0.10)	-1.10 (0.11)	-1.75 (0.13)
Living 4	0.98 (0.10)	0.28 (0.10)	-0.14 (0.10)	-0.38 (0.10)	-0.68 (0.10)	-1.26 (0.11)	-1.91 (0.13)
Living 5	0.87 (0.09)	0.88 (0.10)	0.50 (0.10)	0.32 (0.10)	-0.06 (0.09)	-0.64 (0.10)	-1.21 (0.11)
Safety 1	0.86 (0.10)	0.83 (0.10)	0.39 (0.10)	0.00 (0.09)	-0.41 (0.10)	-0.95 (0.10)	-1.67 (0.12)
Safety 2	1.08 (0.10)	0.70 (0.10)	0.10 (0.10)	-0.22 (0.10)	-0.74 (0.11)	-1.43 (0.12)	-2.12 (0.14)
Safety 3	0.90 (0.10)	1.00 (0.10)	0.49 (0.10)	0.13 (0.10)	-0.27 (0.10)	-0.91 (0.10)	-1.68 (0.12)
Safety 4	1.08 (0.10)	0.97 (0.11)	0.56 (0.10)	0.31 (0.10)	-0.22 (0.10)	-1.01 (0.11)	-1.92 (0.13)
Safety 5	1.09 (0.10)	0.79 (0.11)	0.32 (0.10)	-0.02 (0.10)	-0.53 (0.10)	-1.28 (0.12)	-2.21 (0.14)
Social 1	0.88 (0.10)	0.65 (0.10)	0.25 (0.10)	-0.16 (0.10)	-0.54 (0.10)	-1.25 (0.11)	-2.08 (0.13)
Social 2	0.98 (0.10)	0.90 (0.10)	0.42 (0.10)	0.03 (0.10)	-0.48 (0.10)	-1.27 (0.11)	-2.09 (0.14)
Social 3	0.95 (0.10)	0.72 (0.10)	0.19 (0.10)	-0.11 (0.10)	-0.48 (0.10)	-1.46 (0.12)	-2.32 (0.14)
Social 4	0.69 (0.09)	0.44 (0.09)	0.00 (0.09)	-0.40 (0.09)	-0.96 (0.10)	-1.62 (0.12)	-2.48 (0.15)

Table 5: EAP for sum-score estimates for an eight-item short-form constructed using the largest $\hat{a}^*$ values in Table 4.

Sum-Score	$\hat{\theta}_{EAP}$	$SE(\hat{\theta}_{EAP})$	Sum-Score	$\hat{\theta}_{EAP}$	$SE(\hat{\theta}_{EAP})$
8	-2.001	0.612	33	0.232	0.465
9	-1.668	0.558	34	0.299	0.465
10	-1.533	0.554	35	0.367	0.466
11	-1.447	0.556	36	0.436	0.467
12	-1.388	0.561	37	0.504	0.468
13	-1.285	0.551	38	0.573	0.470
14	-1.213	0.552	39	0.643	0.471
15	-1.084	0.524	40	0.715	0.473
16	-0.999	0.519	41	0.788	0.476
17	-0.920	0.515	42	0.862	0.480
18	-0.843	0.511	43	0.936	0.483
19	-0.760	0.504	44	1.013	0.487
20	-0.682	0.498	45	1.089	0.488
21	-0.603	0.490	46	1.172	0.492
22	-0.529	0.487	47	1.260	0.498
23	-0.457	0.483	48	1.351	0.506
24	-0.385	0.480	49	1.442	0.513
25	-0.314	0.476	50	1.538	0.525
26	-0.244	0.473	51	1.611	0.510
27	-0.174	0.471	52	1.723	0.510
28	-0.106	0.469	53	1.859	0.515
29	-0.038	0.468	54	2.022	0.527
30	0.030	0.467	55	2.205	0.537
31	0.097	0.466	56	2.510	0.579
32	0.165	0.465			

References

Gibbons, R. D., Darrell, R. B., Hedeker, D., Weiss, D. J., Segawa, E., Bhaumik, D. K., … Stover, A. (2007). Full-information item bifactor analysis of graded response data. *Applied Psychological Measurement*, *31*(1), 4–19.
